# Effectiveness of telerehabilitation based on real-time intervention between therapist and participants for improving physical function, activities of daily living and quality of life in people with stroke: A systematic review protocol

**DOI:** 10.1371/journal.pone.0297649

**Published:** 2024-04-02

**Authors:** Ren Fujii, Takahiro Miki, Yuki Nonaka, Shinichiro Tanaka

**Affiliations:** 1 Musashigaoka Clinical Research Center, Musashigaoka Hospital (Tanakakai Medical Corp.), Kumamoto, Japan; 2 Department of Rehabilitation, Musashigaoka Hospital (Tanakakai Medical Corp.), Kumamoto, Japan; 3 Prevent Inc., Nagoya, Aichi, Japan; 4 Department of Rehabilitation Medicine, Musashigaoka Hospital (Tanakakai Medical Corp.), Kumamoto, Japan; New Jersey Institute of Technology, UNITED STATES

## Abstract

**Background:**

There is a call for gathering more evidence on the effectiveness of telerehabilitation in stroke. In particular, a previous systematic review reported substantial variability in the types of technologies used in telerehabilitation interventions. The purpose of this study will be to summarize and synthesize findings on the effects of telerehabilitation based on real-time intervention between therapist and participants for patients with stroke.

**Methods and analysis:**

This systematic review will follow the Preferred Reporting Items for Systematic Review and Meta-Analyses (PRISMA) statement. This systematic review protocol was registered in the International Prospective Register of Systematic Reviews (PROSPERO) on 25 May 2023 (registration number: CRD420234265527). Electronic searches will be performed in the following databases: MEDLINE, Pubmed, Web of Science, PsycINFO and CINAHL electronic databases, using a date range from inception to November 2023. We will include only randomized controlled trials for patients diagnosed with stroke who received telerehabilitation based on real-time interaction between therapist and patients. The exploration will be restricted to publications in the English language. Physical function, activities of daily living and quality of life are the outcomes. We will examine the changes of the outcomes at baseline, at the end of the intervention, and at specific time points during the follow-up after the intervention.

**Discussion:**

This systematic review will provide evidence regarding telerehabilitation for people with stroke.

## Introduction

Stroke is a global health concern that imposes limitations on daily activities [1]. It presents with motor, sensory, cognitive, and mental deficits, diminishing quality of life [2]. Recovery rates vary, with some individuals needing prolonged assistance [3]. Thus, stroke rehabilitation is crucial for both acute recovery and long-term care.

Responding to this need, advancements in information and communication technology have led to the development of telerehabilitation (TR) [4]. This innovative approach allows chronically ill patients, especially stroke survivors, to undergo rehabilitation at home, bridging the gap for those who require sustained intervention [5]. TR employs devices such as smartphones and computers to facilitate therapy without physical contact between healthcare professionals and patients [5]. Research has consistently shown that TR rivals the efficacy of face-to-face sessions in enhancing physical function and daily living activities for stroke patients [6–8]. Moreover, high satisfaction rates reported among TR participants underscore its potential as a practical alternative for stroke rehabilitation, particularly in remote or underserved areas where medical access is limited [9].

TR is promising but must overcome some major challenges. The main issue is that there are no set rules for how to conduct TR [10]. This means that different individuals and institutions will adopt a range of technologies and methods, making it hard for healthcare workers to provide a standardized experience of TR [11]. Technical issues and high costs are also important challenges. In particular, studies focused on the real-time interaction between therapists and patients could greatly improve the effectiveness of TR, leading it to be more widely adopted and trusted as a therapeutic modality.

### Study rationale

Several systematic reviews have examined the impacts of TR [10, 12, 13]. For example, one of these reviews provided the effectiveness of TR for patients with stroke [10]. However, this report also included interventions in the absence of a therapists, such as exercise videos through an electronic tablet, virtual training programs, exercise from a digital video disc, and the combination of physical exercise programs through the internet and gamification [10]. In other words, the types of TR techniques vary considerably, and no specific definitions have been established. The key components of rehabilitation include not only physical training, but also the interactions between the therapist and the participants, such as goal setting based on shared decision making [14]. Therefore, it could be important that the therapist interacts with the participants in real-time using a telecommunication device (e.g., telephone, videophone, and audio-video conference) in TR.

On the other hand, another study has reported the effects of TR using direct video or voice telecommunication, though it encompasses articles published solely up to the year 2017 [12]. The domain of TR should be updated with the most recent articles, considering the numerous studies published in recent years.

Based on the above, in order to establish a methodology of TR, it is necessary to focus on real-time intervention between the therapist and participants and investigate the effects of TR. In addition, stricter inclusion criteria for types of intervention may lead to a more definitive demonstration of the effectiveness of TR. This study aims to summarize and synthesize the impacts of TR based on real-time intervention between therapist and participants for patients with stroke.

### Review objectives

- To summarize the outcomes of TR based on real-time intervention between therapist and participants for patients with stroke.
- To synthesize the evidence on real-time intervention between healthcare professionals and stroke patients.

## Methods

### Protocol and registration

This systematic review was preregistered in the PROSPERO database (registration number: CRD420234265527). The review methodology will follow the guidelines established by the Preferred Reporting Items for Systematic Reviews and Meta-Analysis Protocols (PRISMA-P) [15, 16]. The systematic review results will be reported according to Preferred Reporting Items for Systematic Review and Meta-Analysis (PRISMA 2020 statement) [17].

### Eligibility criteria

Participants will include individuals over 20 years of age who have been diagnosed with a stroke who received a TR based on real-time interaction between therapist and patients.

#### Interventions

TR based on real-time interaction between therapist and patients will be defined as home-based when the real-time interaction takes place via telephone or videoconferencing. We plan to specify that the therapist must use real-time interaction for monitoring physical home exercise, goal settings, or overall treatment.

#### Comparators

In our study, the control group will consist of participants who received rehabilitation interventions that did not incorporate technological tools, such as traditional in-person physical rehabilitation, being placed on a wait-list, or receiving no treatment at all.

#### Outcomes

The outcomes will be physical function (e.g., upper and lower extremity function, balance, gait, physical activity), activities of daily living (e.g., Barthel index and Functional Independence Measure) and quality of life (e.g., EuroQol 5-dimensions 5-levels and Short-Form Health Survey-36). Assessments of these outcomes will be made at baseline, at the end of the intervention, and at specific time points over the post-intervention follow-up.

#### Studies

We will limit our selection to randomized controlled trials exclusively.

#### Publications

Our inclusion criteria are restricted to articles published in the English language. Furthermore, we will concentrate solely on formally published articles, explicitly excluding grey literature from our review.

### Search strategy for identification of relevant studies

Our search will encompass electronic databases such as MEDLINE, Pubmed, Web of Science, PsycINFO and CINAHL, with a focus on literature spanning from their inception up to November 2023. The search strategy was designed according to previous studies [10] and with the assistance of an experienced researcher. The search strategy (S1 File) was composed of blocks of key terms related to the target population, telerehabilitation, outcome, and study design. Furthermore, we plan to conduct a manual examination of relevant literature cited within the studies we identify.

### Study selection and data selection process

The authors, RF and YN, will independently carry out the screening process and detailed analysis of the full text, without concealing details like the publisher, author, or year of publication. In cases of disagreement, another author, TM, will serve as a mediator. Following this thorough evaluation of the full texts, the articles that meet our criteria will be selected, and a flow diagram will be created in accordance with the PRISMA checklist.

### Assessment of methodological quality

The evaluation of argument quality in our study will be conducted using the PEDro score, a specifically designed instrument for assessing the methodological quality [18, 19]. The established score will be presented for comparative analysis. If no score is available, two independent reviewers (RF and YN) will assign a score. Any disagreements will be resolved through mediation by a third author, TM). We will provide a summary of the risk of bias (RoB) for each outcome in each individual study and furnish a table detailing the RoB. PEDro score of ≥6 was classified as moderate-quality to high-quality studies [18].

### Data extraction

The data extraction and entry into a Microsoft Excel spreadsheet will be carried out by a single reviewer, RF. The forms will include (1) first author and year of publication; (2) study types; (3) study design; (4) settings and mode of administration (such as telephone, videoconferencing, and sensors); (5) length of intervention; (6) sample size at the beginning and end of the study; (7) age group; (8) analysis; (9) main results or findings; and (10) reporting of adverse events.

### Data synthesis and analysis

A narrative synthesis will be carried out, which will provide texts and tables to synthesize and discuss the data of the studies and methodological characteristics, as previously described in the data extraction section.

## Discussion

In this systematic review, we will investigate the impacts of TR based on real-time intervention between therapist and participants for patients with stroke. In previous decades, TR has been developed primarily for use in stroke rehabilitation. In fact, a systematic review by Rintala et al. suggested that the effectiveness of technology-based telerehabilitation might be similar to that of traditional treatments in terms of improving physical functioning in stroke patients [10]. However, several limitations exist in this earlier review, with the most important being that substantial variability was observed in the types of technologies in the TR interventions. As a result of this variability, the results of this systematic review should be interpreted with caution. With this background, there is a call for gathering more evidence on the effectiveness of TR in stroke [20]. This review will help to summarize the evidence on TR for stroke patients, as well as support the future development of rehabilitation.

Our protocol has several limitations that bear mention. Specifically, the search will be limited to English-language articles, only published articles will be considered, and the search engine is limited. However, these points are also critical for removing unreliable information. At the same time, the review will have some notable strength. It search strategy and inclusion and exclusion criteria will be systematic, clear, and replicable, as will the approach used to search, screen and extract data. In addition, the involvement of two reviewers in all stages from the selection to the data extraction phase, as well as the assessment of the methodological quality of the included studies, will enhance the methodological rigor and credibility of the results found.

## Supporting information

S1 Checklist PRISMA-P (Preferred Reporting Items for Systematic review and Meta-Analysis Protocols) 2015 checklist: Recommended items to address in a systematic review protocol*. (DOC)

S1 File Search strategy. (DOCX)
