# Supplementary material for: Effectiveness of telerehabilitation based on real-time intervention between therapist and participants for improving physical function, activities of daily living and quality of life in people with stroke: A systematic review protocol
Source: PLoS One. 2024 Apr 2;19(4):e0297649. doi: 10.1371/journal.pone.0297649 (PMC10986948; doi:10.1371/journal.pone.0297649)
Supplement: S1 File — (DOCX) [file pone.0297649.s002.docx]

**Search Strategy**

| 1 | exp stroke/ |
| --- | --- |
| 2 | poststroke$.ti,ab. |
| 3 | stroke$.ti,ab. |
| 4 | apoplexy.ti,ab. |
| 5 | Hemiplegia/ |
| 6 | hemiplegia$.ti,ab. |
| 7 | monoplegia$.ti,ab. |
| 8 | Brain Ischemia/ |
| 9 | brain ischemia$.ti,ab. |
| 10 | cerebral ischemia$.ti,ab. |
| 11 | brain infarction$.ti,ab. |
| 12 | Cerebrovascular Disorders/ |
| 13 | cerebrovascular dis$.ti,ab. |
| 14 | intracranial vascular dis$.ti,ab. |
| 15 | cerebrovascular occlusion$.ti,ab. |
| 16 | cerebrovascular insufficien$.ti,ab. |
| 17 | cerebrovascular accident$.ti,ab. |
| 18 | 1 or 2 or 3 or 4 or 5 or 6 or 7 or 8 or 9 or 10 or 11 or 12 or 13 or 14 or 15 or 16 or 17 |
| 19 | telerehabilitation/ |
| 20 | telerehabilitation$.ti,ab. |
| 21 | tele rehabilitation$.ti,ab. |
| 22 | remote rehabilitation$.ti,ab. |
| 23 | telemedicine/ |
| 24 | telemedicine$.ti,ab. |
| 25 | tele medicine$.ti,ab. |
| 26 | telehealth$.ti,ab. |
| 27 | ehealth$.ti,ab. |
| 28 | mobile health$.ti,ab. |
| 29 | mhealth$.ti,ab. |
| 30 | phealth$.ti,ab. |
| 31 | Remote Consultation/ |
| 32 | online meeting$.ti,ab. |
| 33 | Videoconferencing/ |
| 34 | videoconferenc$.ti,ab. |
| 35 | video conferenc$.ti,ab. |
| 36 | zoom.ti,ab. |
| 37 | microsoft teams.ti,ab. |
| 38 | Skype.ti,ab. |
| 39 | webex.ti,ab. |
| 40 | google meet.ti,ab. |
| 41 | 19 or 20 or 21 or 22 or 23 or 24 or 25 or 26 or 27 or 28 or 29 or 30 or 31 or 32 or 33 or 34 or 35 or 36 or 37 or 38 or 39 or 40 |
| 42 | Physical Therapists/ |
| 43 | physiotherapist$.ti,ab. |
| 44 | Occupational Therapists/ |
| 45 | therapist$.ti,ab. |
| 46 | Patient Education as Topic/ |
| 47 | patient education$.ti,ab. |
| 48 | Behavior Therapy/ |
| 49 | behavior therap$.ti,ab. |
| 50 | Cognitive Therapy/ |
| 51 | cognitive therap$.ti,ab. |
| 52 | Cognitive Behavioral Therapy/ |
| 53 | Exercise Therapy/ |
| 54 | exercise therap$.ti,ab. |
| 55 | Physical Therapy Modalities/ |
| 56 | physical therap$.ti,ab. |
| 57 | physiotherap$.ti,ab. |
| 58 | Neurophysiotherap$.ti,ab. |
| 59 | Occupational Therapy/ |
| 60 | occupational therap$.ti,ab. |
| 61 | ergotherap$.ti,ab. |
| 62 | functional therap$.ti,ab. |
| 63 | Rehabilitation/ |
| 64 | rehabilitation$.ti,ab. |
| 65 | stroke rehabilitation/ |
| 66 | Communication/ |
| 67 | communication$.ti,ab. |
| 68 | Physical Functional Performance/ |
| 69 | Physical Performance$.ti,ab. |
| 70 | Functional Performance$.ti,ab. |
| 71 | Exercise/ |
| 72 | exercise.ti,ab. |
| 73 | physical activit$.ti,ab. |
| 74 | Exercise Movement Techniques/ |
| 75 | Motor Activity/ |
| 76 | motor activit$.ti,ab. |
| 77 | Activities of Daily Living/ |
| 78 | ("activities of daily living" or "activities of daily livings").ti,ab. |
| 79 | Quality of Life/ |
| 80 | quality of life.ti,ab. |
| 81 | life qualit$.ti,ab. |
| 82 | 42 or 43 or 44 or 45 or 46 or 47 or 48 or 49 or 50 or 51 or 52 or 53 or 54 or 55 or 56 or 57 or 58 or 59 or 60 or 61 or 62 or 63 or 64 or 65 or 66 or 67 or 68 or 69 or 70 or 71 or 72 or 73 or 74 or 75 or 76 or 77 or 78 or 79 or 80 or 81 |
| 83 | Randomized Controlled Trials as Topic/ |
| 84 | Randomized Controlled Trial/ |
| 85 | randomi#ed controlled trial$.pt,ab. |
| 86 | 83 or 84 or 85 |
| 87 | 18 and 41 and 82 and 86 |
